# Factors associated with postoperative efficacy evaluation in patients with morbid obesity

**DOI:** 10.1038/s41598-024-63099-4

**Published:** 2024-05-28

**Authors:** Tai-Hsiang Chen, Wen-Wen Huang, Liu-Chun Lu, Chen-Chung Ma

**Affiliations:** 1 Department of Healthcare Administration, Linkou Chang Gung Memorial Hospital, Taoyuan, Taiwan, R.O.C.; 2 Executive Master Program of Healthcare Administration, I-Shou University, Kaohsiung, Taiwan, R.O.C.; 3 Operating Room, E-DA Dachang Hospital, Kaohsiung, Taiwan, R.O.C.; 4 Department of Healthcare Administration, I-Shou University, Kaohsiung, Taiwan, R.O.C.

**Keywords:** Morbid obesity, Quality of care, Complications, Obesity management, Risk control, Health care, Risk factors

## Abstract

The global obesity problem is becoming increasingly serious, with eight of the top ten causes of death in Taiwan in 2020 being related to obesity. Morbid obesity poses a significant threat to one’s health and well-being. In recent years, bariatric surgery has emerged as a more effective treatment option for patients with morbid obesity. However, the procedure is not without risks. This study aims to examine the factors that impact the postoperative efficacy evaluation of patients with morbid obesity. This study uses a retrospective cross-sectional design, with medical records being collected retrospectively. The data was collected from patients who underwent bariatric surgery between July 1, 2017 and June 30, 2020 at a hospital in southern Taiwan. A total of 663 patients were included in the study and were observed for 1 year after the surgery. The independent variables included demographic variables, perceived symptoms variables, perceived lifestyle variables, and surgery-related variables, while the dependent variables included weight loss outcomes and complications. The prognostic factors affecting the postoperative efficacy evaluation of patients with pathological obesity were determined using multiple regression analysis and binary regression analysis. The study found that 65.6% of the participants were female, with an average age of 36.8 years. The results of the multiple regression and binary logistic regression showed that gender, age, BMI, diabetes, and smoking habit were the predictors of postoperative weight loss. Hypertension, diabetes, liver disease, kidney disease, smoking habit, drinking habit, and operation time were the predictors of postoperative complications. The study found that the presence of the aforementioned 12 significant factors can affect the success of weight loss after surgery and the incidence of postoperative complications. This information can serve as a reference for clinical care institutions and patients to improve the postoperative efficacy evaluation.

## Introduction

As the world evolves and experiences changes in the environment, diet, and lifestyle, as well as a prolonged period of sedentary work, obesity has become a global concern. The World Health Organization reported in 2020 that there were approximately 650 million people aged 18 and above who were considered obese. Moreover, according to the same report, an alarming 38 million children under the age of 5 were either overweight or obese in 2019^1^. Additionally, the Health Promotion Administration of the Ministry of Health and Welfare reported that eight out of the ten leading causes of death in 2020 were related to obesity, including heart disease, stroke, diabetes, chronic lower respiratory diseases, hypertension, cancer, chronic liver disease, and chronic kidney disease^2^. To treat obesity, various medical methods are currently available, including dietary and nutrition guidance, drug therapy, surgical treatment, and physical exercise. However, it has been found that the efficacy of dietary and drug therapies is limited, with a success rate of less than 5% for patients with morbid obesity. As a result, surgical treatment has become the preferred option for those with morbid obesity.

In recent years, the proportion of patients receiving bariatric surgery in Taiwan has steadily increased, and most people believe this method can be an effective means of weight loss. However, the postoperative effects of different bariatric surgery methods vary. Currently, weight loss surgery is divided into several types: sleeve gastrectomy, Roux-en-Y Gastric Bypass, Adjustable Gastric Banding, and BioEnterics Intragastric Balloon. Among these, Roux-en-Y Gastric Bypass has the best outcome, while laparoscopic operation is the most widely accepted by patients^3,4^. According to the 2018 American Society for Metabolic and Bariatric Surgery, patients with morbid obesity can expect to lose up to 60% of their excess body weight in the six months following bariatric surgery. In some cases, as much as 77% of excess body weight can be lost within the first year post-surgery^5^. Patients with a BMI of less than 40 before surgery are more likely to have a BMI of less than 30 after surgery and see remission of obesity-related illnesses, compared to those who did not undergo surgery^6^.

With changes in diet, lifestyle, and technological development and innovation, obesity has become a leading contributor to chronic illnesses. Obesity can cause harm not only to the body and mind, but also to one’s health, with the health risk for patients with morbid obesity being twice that of normal people. In today’s society, people seek good posture and health, leading to a wide variety of weight loss methods. Incorrect weight loss methods, such as unbalanced diets, taking weight-loss drugs prescribed by non-physicians, and even taking appetite suppressants, can cause physical harm and result in even more health issues^7^. This study aimed to investigate the factors that affect the efficacy evaluation of patients with morbid obesity after surgery. Specifically, the study aimed to explore the correlation between demographic variables (age, gender, body mass index), perceived symptom variables which means an individual’s self-perception or assessment of his or her own disease (hypertension, heart disease, diabetes, liver disease, kidney disease), perceived lifestyle variables (smoking habits, drinking habits), surgery-related variables (surgical procedure, surgical duration), and postoperative efficacy evaluation variables (complications, weight loss). Based on the findings, weight loss centers and clinical care can provide preoperative reference to improve the health outcomes of patients with morbid obesity and enhance the safety of surgical care.

## Methodology

### Sampling and data collection

The independent variables are demographic variables including gender, age, and body mass index (BMI). Gender is divided into male and female, age is a continuous variable, and BMI is classified according to reference literature into BMI less than or equal to 40 and BMI greater than 40^6^. The perceived symptom variables include hypertension, heart disease, diabetes, liver disease, and kidney disease, all divided into present or absent. Heart disease is defined as cardiovascular disease, hereditary or congenital heart disease, rheumatic heart disease, viral heart disease, or coronary heart disease as diagnosed by a physician. Kidney disease is defined as a progressive loss of kidney function and an inability to filter excess fluid and water from the body. The perceived lifestyle variables include smoking and drinking habits, both divided into present or absent. Surgery-related variables include surgical techniques and surgical time. The surgical techniques are divided into two groups: sleeve gastrectomy and other techniques, and surgical time is a continuous variable. The dependent variable is postoperative efficacy evaluation, including weight loss efficacy and complications. In terms of weight loss efficacy, according to the Health and Welfare Ministry of Taiwan’s Food Information Website, the ideal body mass index (BMI) is 22. An effective method for evaluating weight loss surgery efficacy is the Excess Body Weight Loss Percentage (%EWL)^8,9^. In terms of complications, it is divided into present or absent. If patients experience infections, hernias, gastroesophageal reflux, and even abnormal immune system functions, pulmonary adjustment dysfunction, reoperation, or death after the surgery, they are considered to have complications^4^.

### Measures

In order to ensure data consistency, random sampling was conducted according to the method suggested by scholars^10^, and 10% patient data were randomly selected, a total of 66 samples, and data verification was carried out in the same way and with the same data. After verification, there were 7 case data login errors in this study, and the accuracy of case data was 89.39%. After comparing the wrong data according to the verification results, the researcher logged in the case data again. In order to improve the consistency of data extraction, 10% patient data were randomly selected in the same way, a total of 66 samples. After the second verification, the extracted data was correct and the consistency reached 100%. In order to ensure that the privacy of patients is not violated, the patient’s medical record number and name are linked before the relevant analysis, so that the patient’s data cannot be searched and identified.

### Analysis

In this study, IBM SPSS Statistics 25 was used for statistical analysis, and α = 0.05 was used for all tests. Descriptive and inferential statistics were performed according to the research purpose and structure. Descriptive statistics are represented by frequency distribution table, percentage, mean and standard deviation. Based on the properties of each variable, inferential statistics include independent t-test, Chi-Squared Test, Multiple regression analysis and Logistic regression (logit model) were used to identify the factors influencing postoperative efficacy evaluation of patients with morbid obesity.

### Ethical approval

The institutional review board (IRB) of the E-DA hospital approved this study (EMRP-110-045). This study does not involve live animals and individual human participants. The requirement for informed consent from the study subjects was waived by the IRB of the E-DA hospital due to the reason that the data analyzed in this study are de-identification, anonymized, and aggregated within the hospital. No personally identifiable information, such as an individual’s location, contacts, or movement, was made available at any point. This study confirms that all methods were performed in accordance with the relevant guidelines and regulations. The datasets analyzed during the current study are not publicly available due privacy of the patients but are available from the corresponding author on reasonable request.

## Results

The results showed that among the 663 bariatric surgery patients, 435 (65.6%) were mostly female. The mean age was 36.88 ± 10.25 years, and the body mass index (BMI) ranged from 32.5 to 40, with a total of 450 (67.9%). The number of perceived symptom variables were greater in those without hypertension (51.0%) than in those with hypertension (49.0%), and in those without heart disease (94.9%) than in those with heart disease (5.1%). Those without diabetes (82.8%) were greater than those with diabetes (17.2%), those with liver disease (68.8%) were greater than those without liver disease (31.2%), and those without kidney disease (78.7%) were greater than those with kidney disease (21.3%). Among the perceived lifestyle variables, those without smoking habits (76.8%) were greater than those with smoking habits, and those without drinking habits (87.5%) were greater than those with drinking habits (12.5%). Surgery-related variables were more common among sleeve gastrectomy patients (98.3%), and the average surgery time was 81.22 ± 18.05 min. The data were compiled in Table 1.

**Table 1:** Descriptive statistical results in patients with morbid obesity (n = 663).

Variables	Female (%) (n = 435)	Male (%) (n = 228)
Age	36.57 ± 10.04	37.14 ± 9.54
Body mass index (BMI)		
32.5 < BMI < 40	315 (72.4)	135 (59.2)
BMI ≧ 40	120 (27.6)	93 (40.8)
Hypertension		
No	222 (51.0)	103 (45.2)
Yes	213 (49.0)	125 (54.8)
Heart disease		
No	414 (95.2)	215 (94.3)
Yes	21 (4.8)	13 (5.7)
Diabetes		
No	365 (83.9)	184 (80.7)
Yes	70 (16.1)	44 (19.3)
Liver disease		
No	123 (28.3)	84 (36.8)
Yes	312 (71.7)	144 (63.2)
Kidney disease		
No	368 (84.6)	154 (67.5)
Yes	67 (15.4)	74 (32.5)
Smoking habit		
No	363 (83.4)	146 (64.0)
Yes	72 (16.6)	82 (36.0)
Drinking habit		
No	411 (94.5)	169 (74.1)
Yes	24 (5.5)	59 (25.9)
Surgical method		
Sleeve gastrectomy	429 (98.6)	223 (97.8)
Other operations	6 (1.4)	5 (2.2)
Operation time	79.27 ± 16.80	84.94 ± 19.73

The independent sample T-test results of weight loss results for demographic variables (gender, body mass index), perceived symptom variables, perceived lifestyle variables, surgery-related variables (surgical procedure) and postoperative efficacy evaluation variables are shown in Table 2. In terms of gender (t = 3.351, p < 0.001), the results of weight loss after surgery were statistically significant, indicating that the effect of female was higher than that of male. There were statistically significant differences between body mass index (t = 9.101, p < 0.001) and postoperative weight loss, indicating that patients with body mass index between 32.5 and 40 kg/m^2^ had higher weight loss than those with body mass index greater than 40. In terms of perceived symptom variables, diabetes (t = 3.866, p < 0.001) and weight loss results showed a statistically significant difference, indicating that the weight loss effect of patients without diabetes was higher than that of patients with diabetes. The data are shown in Table 2.

**Table 2:** Independent sample T-test of variables and postoperative weight loss in patients with morbid obesity (n = 663).

Variables	Number	M ± SD	t value	p value
Gender				
Female	435	82.05 ± 20.98	3.351	< 0.001
Male	228	76.36 ± 20.43		
Body mass index (BMI)				
32.5 < BMI < 40	450	84.58 ± 21.18	9.101	< 0.001
BMI ≧ 40	213	70.62 ± 16.98		
Hypertension				
No	338	80.32 ± 20.62	0.276	0.783
Yes	325	79.87 ± 21.30		
Heart disease				
No	629	80.00 ± 20.98	− 0.508	0.612
Yes	34	81.87 ± 20.63		
Diabetes				
No	549	81.51 ± 20.54	3.866	< 0.001
Yes	114	73.26 ± 21.66		
Liver disease				
No	207	79.46 ± 20.62	− 0.526	0.599
Yes	456	80.38 ± 21.12		
Kidney disease				
No	522	79.72 ± 20.71	− 0.875	0.382
Yes	141	81.46 ± 21.84		
Smoking habit				
No	509	79.64 ± 20.86	− 1.013	0.311
Yes	154	81.59 ± 21.24		
Drinking habit				
No	580	80.63 ± 20.69	1.729	0.084
Yes	83	76.38 ± 22.47		
Surgical method				
Sleeve gastrectomy	652	80.26 ± 21.01	1.535	0.125
Other operations	11	70.48 ± 14.99		

The chi-square test results of demographic variables (gender, body mass index), perceived symptom variables, perceived lifestyle variables, surgery-related variables (surgical procedures) and postoperative efficacy evaluation variables are shown in Table 3. The results showed that gender (χ^2^ = 12.245, p < 0.001), hypertension (χ^2^ = 83.119, p < 0.001), heart disease (χ^2^ = 6.911, p = 0.009), diabetes (χ^2^ = 38.671, p < 0.001), liver disease (χ^2^ = 66.916, p < 0.001), kidney disease (χ^2^ = 52.816, p < 0.001), smoking habit (χ^2^ = 38.145, p < 0.001), alcohol habit (χ^2^ = 22.469, p < 0.001) 52.816, p < 0.001), smoking habit (χ^2^ = 38.145, p < 0.001), and drinking habit (χ^2^ = 22.469, p < 0.001) showed statistically significant differences on postoperative complications.

**Table 3:** Chi-square test of variables and postoperative complications in morbidly obese patients (n = 663).

Variables	Complication		χ^2^	p value
	Yes (%)	No (%)		
Gender				
Female	232 (35.0)	203 (30.6)	12.245	< 0.001
Male	89 (13.4)	139 (21.0)		
Body mass index (BMI)				
32.5 < BMI < 40	216 (32.6)	234 (35.3)	0.097	0.755
BMI ≧ 40	105 (15.8)	108 (16.3)		
Hypertension				
No	216 (32.6)	109 (16.4)	83.119	< 0.001
Yes	105 (15.8)	233 (35.1)		
Heart disease				
No	312 (47.1)	317 (47.8)	6.911	0.009
Yes	9 (1.4)	25 (3.8)		
Diabetes				
No	296 (44.6)	253 (38.2)	38.671	< 0.001
Yes	25 (3.8)	89 (13.4)		
Liver disease				
No	149 (22.5)	58 (8.7)	66.916	< 0.001
Yes	172 (25.9)	284 (42.8)		
Kidney disease				
No	291 (43.9)	231 (34.8)	52.816	< 0.001
Yes	30 (4.5)	111 (16.7)		
Smoking habit				
No	280 (42.2)	229 (34.5)	38.145	< 0.001
Yes	41 (12.8)	113 (33.0)		
Drinking habit				
No	301 (45.4)	279 (42.1)	22.469	< 0.001
Yes	20 (3.0)	63 (9.5)		
Surgical method				
Sleeve gastrectomy	316 (47.7)	336 (50.7)	0.039	0.843
Other operations	5 (0.8)	6 (0.9)		

In multiple regression analysis, the relationship between variables and weight loss results of postoperative efficacy evaluation. First, the basic test of multiple regression is conducted. When the average of standardized residual value is zero and the standard deviation is 0.99 (within ± 1.96), it shows that the regression line is suitable. The boundary of variance expansion coefficient (VIF) was 1.054–1.955. Since VIF did not exceed 10, it could be seen that there was no collinear situation among the variables. Therefore, the regression model was consistent with the basic assumption of regression analysis. Abstract of the model, this model has 15.5% explanatory power to predict the effect of postoperative weight loss, and the result is significant (F = 11.108, p < 0.001), indicating that this model has predictive power.

In terms of demographic variables, women were used as the reference group. standardized regression coefficient of postoperative weight loss for men was − 0.102, which was statistically significant (p = 0.011), indicating that men had lower weight loss efficiency than women. Age was a continuous variable, and the standardized regression coefficient was − 0.175, which was statistically significant (p < 0.001), indicating that the lower the age, the higher the weight loss effect. For body mass index (BMI), the standardized regression coefficient of 32.5 < body mass index (BMI) < 40 was − 0.313, which was statistically significant (p < 0.001). This suggests that patients with morbid obesity with a body mass index (BMI) of 32.5 to 40 will lose more weight after weight-loss surgery than those with a BMI greater than 40.

The multivariate regression analysis of perceived symptom variables on postoperative weight loss results showed that patients without hypertension were taken as the reference group, and the standardized regression coefficient of postoperative weight loss results in patients with hypertension was 0.005, which was not statistically significant (p = 0.920). In terms of cardiac disease variables, the standardized regression coefficient of postoperative weight loss in patients with cardiac disease was 0.047, which was not statistically significant (p = 0.200). In terms of diabetes variables, patients without diabetes were taken as the reference group. The standardized regression coefficient of postoperative weight loss in patients with diabetes was − 0.126, which was statistically significant (p = 0.001), indicating that patients with diabetes had lower weight loss results than those without diabetes. In terms of liver disease variables, patients without liver disease were used as the reference group. The standardized regression coefficient of postoperative weight loss in patients with liver disease was − 0.017, which was not statistically significant (p = 0.726). In terms of kidney disease variables, patients without kidney disease were used as the reference group. The standardized regression coefficient of postoperative weight loss in patients with kidney disease was 0.073, which was not statistically significant (p = 0.064). The multivariate regression analysis of the effect of perceived lifestyle variables on postoperative weight loss results showed that patients without smoking habits were taken as the reference group in terms of smoking habits variables. The standardized regression coefficient of postoperative weight loss results in patients with smoking habits was 0.109, which was statistically significant (p = 0.006). These results indicated that smokers had higher weight loss results after weight loss surgery than those without smoking habit. Patients without drinking habits were taken as the reference group, and the standardized regression coefficient of postoperative weight loss effect of patients with drinking habits was − 0.059, which was not statistically significant (p = 0.142). Multiple regression analysis of surgery-related variables on postoperative weight loss results. In terms of surgical variables, patients undergoing sleeve gastrectomy were selected as the reference group. The standardized regression coefficient of postoperative weight loss results in patients undergoing other surgical procedures was − 0.008, which was not statistically significant (p = 0.834). The operation time was a continuous variable, and the standardized regression coefficient was 0.006, which was not statistically significant (p = 0.876). The above results were shown in Table 4.

**Table 4:** Results of multiple regression analysis of postoperative weight loss in patients with morbid obesity (n = 663).

Variables	Beta	p value	F value	R^2^	Adjusted R^2^
Model			11.108	17.0	15.5
Constant		< 0.001			
Gender					
Female (reference)					
Male	− 0.102	0.011*			
Age	− 0.175	< 0.001***			
Body mass index					
32.5 < BMI < 40 (reference)					
BMI ≧ 40	− 0.313	< 0.001***			
Hypertension					
No (reference)					
Yes	0.005	0.920			
Heart disease					
No (reference)					
Yes	0.047	0.200			
Diabetes					
No (reference)					
Yes	− 0.126	0.001**			
Liver disease					
No (reference)					
Yes	− 0.017	0.726			
Kidney disease					
No (reference)					
Yes	0.073	0.064			
Smoking habit					
No (reference)					
Yes	0.109	0.006**			
Drinking habit					
No (reference)					
Yes	− 0.059	0.142			
Surgical method					
Sleeve gastrectomy (reference)					
Other operations	− 0.008	0.834			
Operation time	0.006	0.876			

*p < 0.05, **p < 0.01, ***p < 0.001.

In terms of the statistical results of binary logistic regression analysis on complications of postoperative efficacy evaluation, Omnibus was first used to verify whether the model was representative, and the fitness of the model of the self-variable terms of this study was tested by binary logistic regression, and the results showed significant (p < 0.001). It can be expressed that at least one of the selected self-variable terms can predict the probability of complications in the outcome variable. The next is the difference between the number of observations and the expected number, including all the predicted variables. Hosmer and Lemeshow was used to detect the fit statistics of the model, and the test result was no statistically significant difference (chi-square statistic was 8.747, p = 0.364), indicating that there was no difference between the number of observations and the expected number. It means that the model has a good fit. Among the demographic variables, females were used as the reference group, and Adjusted odds ratio (AOR) for males was 1.066 times of that for females, with no significant difference (p = 0.767). In terms of age, the incidence of postoperative complications increased by 0.994 times if the age increased by one year, with no significant difference (p = 0.541). In terms of body mass index (BMI), patients with 32.5 < body mass index (BMI) < 40 as the reference group, the AOR of 32.5 < body mass index (BMI) < 40 were 0.831 times higher than those with ≧ 40, with no significant difference (p = 0.358). Patients without hypertension were taken as the reference group. The AOR adjusted for postoperative complications was 2.617 times higher in patients with hypertension than in patients without hypertension (p < 0.001, 95% CI 1.648–4.154) as a predictor of postoperative complications in patients with morbid obesity; In terms of cardiac disease variables, the AOR of patients with cardiac disease was 1.647 times higher than that of patients without cardiac disease (p = 0.259). In terms of diabetes variables, patients without diabetes were taken as the reference group. The AOR of patients with diabetes was 2.062 times that of patients without diabetes, showing a significant difference (p = 0.008, 95% CI 1.204–3.533), which was a predictor of postoperative complications in patients with morbid obesity. In terms of liver disease variables, patients with liver disease were taken as the reference group, and the AOR for postoperative complications was 1.968 times that of patients without liver disease, with a significant difference (p = 0.016, 95% CI 1.135–3.413), is a predictor of postoperative complications in patients with morbid obesity; In terms of kidney disease variables, patients with kidney disease were used as the reference group, and the AOR for postoperative complications was 2.811 times that of patients without kidney disease, with a significant difference (p < 0.001, 95% CI 1.702–4.644) is a predictor of postoperative complications in patients with morbid obesity.

In terms of the smoking habit variable, patients with smoking habit were used as the reference group, and the AOR of patients with smoking habit was 3.489 times higher than those without smoking habit, showing a significant difference (p < 0.001, 95% CI 2.136–5.697), is a predictor of postoperative complications in patients with morbid obesity; In terms of the variables of drinking habits, patients without drinking habits were taken as the reference group, and the AOR of patients with drinking habits was 2.770 times that of patients without drinking habits, showing a significant difference (p = 0.002, 95% CI 1.437–5.337) as a predictor of postoperative complications in patients with morbid obesity.

The results of binary logistic regression analysis of postoperative complications for surgery-related variables showed that the AOR for patients undergoing sleeve gastrectomy was 0.864 times higher than that for patients undergoing sleeve gastrectomy, with no significant difference (p = 0.864). In terms of operation time, if the operation time was increased by one minute, the incidence of postoperative complications would increase by 1.014 times, with a significant difference (p = 0.021, 95% CI 1.002–1.026), which is a predictor of postoperative complications in patients with pathological obesity. The data are shown in Table 5.

**Table 5:** Results of binary logistic regression analysis of postoperative complications in morbidly obese patients (n = 663).

Variables	Adjusted odds ratio	95% confidence interval		p value
		Lower	Upper	
Gender				
Female (reference)				
Male	1.066	0.697	1.631	0.767
Age	0.994	0.975	1.013	0.541
Body mass index				
32.5 < BMI < 40 (reference)				
BMI ≧ 40	0.831	0.561	1.233	0.358
Hypertension				
No (reference)				
Yes	2.617	1.648	4.154	< 0.001***
Heart disease				
No (reference)				
Yes	1.647	0.693	3.913	0.259
Diabetes				
No (reference)				
Yes	2.062	1.204	3.533	0.008**
Liver disease				
No (reference)				
Yes	1.968	1.135	3.413	0.016*
Kidney disease				
No (reference)				
Yes	2.811	1.702	4.644	< 0.001***
Smoking habit				
No (reference)				
Yes	3.489	2.136	5.697	< 0.001***
Drinking habit				
No (reference)				
Yes	2.770	1.437	5.337	0.002**
Surgical method				
Sleeve gastrectomy (reference)				
Other operations	0.864	0.161	4.634	0.864
Operation time	1.014	1.002	1.026	0.021*

*p < 0.05, **p < 0.01, ***p < 0.001.

## Discussion

In recent years, patients with morbid obesity are gradually using bariatric surgery as a treatment method. The weight loss effect and complications will affect the postoperative efficacy evaluation and patient safety. Multiple regression analysis showed that gender (β =  − 0.012, p = 0.011), age (β =  − 0.175, p < 0.001) and body mass index (BMI) (p < 0.001) were all predictors of pathological obesity. In terms of gender, the weight loss effect of females was better than that of males, with a statistically significant difference (p < 0.001), which was different from the research of domestic scholars, which showed that the weight loss effect of males was better than that of females. The reason for the difference may be that the number of cases in this study was more female than male. Age was consistent with previous studies, which found a statistically significant difference between age and postoperative weight loss (p < 0.001)^11^. In terms of body mass index (BMI), morbidly obese patients with body mass index (BMI) between 32.5 and 40 will have better weight loss results than those with body mass index (BMI) greater than 40, which is similar to the results of foreign studies that show that body mass index (BMI) less than 40^6,12^.

In terms of perceived symptom variables, diabetes (p = 0.005) showed that patients with diabetes had lower weight loss after weight loss surgery than those without diabetes, with a statistically significant difference (p = 0.005). There was no significant relationship between the percentage of postoperative excess weight loss (% EWL) of patients with diabetes symptoms (p = 0.848)^13^. Among the perceived lifestyle habit variables, smoking habit (p = 0.002), the study results showed that patients with smoking habit had higher weight loss results after weight loss surgery than those without smoking habit. Patients with smoking habits had a significant relationship (p = 0.005) with the percentage of excess weight lost after surgery (% EWL)^13^.

The results of binary logistic regression analysis of complications showed that in hypertensive patients, the AOR of postoperative complications were 2.617 times higher in morbidly obese patients with hypertension than in patients without hypertension (p < 0.001, 95% CI 1.648–4.154), indicating that hypertension is a predictor of postoperative complications, which is similar to the results of Dutch scholars’ study, showing a significant difference between hypertension and postoperative complications in patients undergoing weight loss surgery (p = 0.012, 95% CI 1.076–1.812), and similar to the results of this study^14^. The past study has shown that blood vessel walls in hypertensive patients may become more rigid and fragile, increasing the risk of cardiovascular events after surgery, and fluctuations in blood pressure may impose an additional burden on cardiac and renal function, affecting postoperative recovery^15^. In terms of diabetes, the AOR for postoperative complications in morbidly obese patients with diabetes symptoms were 2.062 times higher than those without diabetes (p = 0.008, 95% CI 1.204–3.533), indicating that diabetes is a predictor of postoperative complications. Studies by American scholars showed that patients with diabetes after weight loss surgery had 0–1 months after surgery (OR 1.3, p = 0.017, 95% CI 1.04–1.50) and 4–6 months after surgery (OR 1.8, p = 0.001, 95% CI 1.31–2.47) had a higher probability of infection, which was similar to this study^16^. The past study has shown that people with diabetes are more prone to infections and have slower wound healing due to poor blood sugar control. High blood sugar levels may also cause damage to blood vessels and affect blood circulation, which can affect recovery after surgery^17^. In terms of liver disease, the AOR for postoperative complications in morbidly obese patients with liver disease symptoms were 1.968 times higher than those without liver disease (p = 0.016, 95% CI 1.135–3.413), indicating that liver disease was a predictor of postoperative complications, which was significantly different from the results of Dutch scholars’ studies, showing that there was a significant difference between the presence of liver disease and postoperative complications in weight-loss surgery patients (OR 1.396, p = 0.042, 95% CI 1.012–1.928). The previous study has shown that poor liver function affects drug metabolism and blood clotting, increasing the risk of postoperative bleeding and infection. The nutritional status of people with liver disease may also be poor, further affecting the recovery process^18^. Moreover, the Kidney disease of AOR for postoperative complications in morbidly obese patients with liver disease symptoms were 2.811 times higher than those without liver disease (p ≤ 0.001, 95% CI 1.702–4.644). The previous study has also shown that renal dysfunction can affect fluid and electrolyte balance in the body, increase the risk of cardiovascular disease and infection after surgery, and may require adjustment of medication dosages to avoid drug accumulation and toxicity^19^. Similar to the results of this study^14^, since both this study and foreign literature have found that patients suffering from hypertension, diabetes, liver disease or kidney disease may increase the incidence of postoperative complications, nursing units can be provided as the basis for preoperative evaluation.

For the perceived lifestyle variables, the AOR for postoperative complications were 3.489 times higher in patients with smoking habit symptoms than in patients without smoking habit (p < 0.001, 95% CI 2.136–5.697), indicating that smoking habit is a predictor of postoperative complications, which is statistically significant different from American scholars’ studies showing that smoking habit and postoperative complications in weight-loss surgery patients (OR 1.2, p = 0.03), which is similar to the results of this study^20^. The past study indicated that smoking affects lung function and oxygenation, increasing the risk of postoperative respiratory complications such as lung infections and respiratory failure. Smoking also slows wound healing and increases the risk of infection^21^. The AOR for postoperative complications in morbidly obese patients with drinking habits were 2.770 times higher than those without drinking habits (p = 0.009, 95% CI 1.437–5.337), indicating that drinking habit is a predictor of postoperative complications, which is statistically significantly different from that of American scholars (OR 1.55, p = 0.009), which is similar to the results of this study^22^. These results found that smoking or drinking habits may increase the risk of postoperative complications. The past study has suggested that chronic alcohol use may lead to liver insufficiency and malnutrition, increase the risk of bleeding after surgery, and affect drug metabolism^23^.

Among surgery-related variables, the AOR for postoperative complications in patients with morbid obesity in terms of surgical time spent performing bariatric surgery was 1.014 (p = 0.021, 95% CI 1.002–1.026), indicating that operation time was a predictor of postoperative complications, which was statistically significant different from that of American scholars (OR 1.27 p < 0.001, 95% CI (1.19, 1.35)). Similar to the results of this study^24^, since both foreign studies and the results of this study found that the duration of surgery may increase the risk of complications, the study results can be provided to weight loss centers and clinical care related units as relevant evidence before surgery. The past study suggested that longer surgery times may mean that the procedure is more complex, more physically demanding and tolerable to the patient, and that prolonged anesthesia and surgical procedures may increase the risk of postoperative complications, including thrombosis, infection, and prolonged recovery time^25^.

## Conclusions

This study found that gender, age, BMI, diabetes and smoking habits were predictors of postoperative weight loss. Hypertension, diabetes, liver disease, kidney disease, smoking, alcohol consumption, and operation time were predictors of postoperative complications. If the patient has symptoms related to these 12 significant factors, it will affect the outcome of postoperative weight loss and the occurrence of complications. To provide reference for clinical nursing institutions and patients to improve postoperative efficacy evaluation. In terms of research limitations, secondary databases were used in this study, and only individual hospital data could be used for analysis and discussion of relevant variables. Due to certain risks of cancer diseases for weight-loss surgery, cancer-related data could not be extracted due to limited database integrity. In addition, according to a 2018 report from the World Health Organization (WHO), the quality of life of morbidly obese patients may also be affected by physical and psychological changes. As this study used secondary databases to conduct research, the integrity of the databases was limited, and the nursing data related to length of hospital stay and quality of life could not be extracted. It is also due to the retrospective design of medical records used in the study, only basic personal data such as height, weight, chronic diseases and living habits were collected for the this study. There is no questionnaire related data on nutrition and quality of life after surgery. This study was designed as a retrospective cross-sectional study, unlike longitudinal studies that tracked weight loss before and after surgery. Since the sample in this study only presents a regional hospital, the research results may not be applied to all hospitals due to the limited number of samples. It is expected to expand the study sample size for future researchers reference.

## Data Availability

The datasets analysed during the current study are not publicly available due privacy of the patients but are available from the corresponding author on reasonable request.
